# Large Language Models Perform at Chance Level in the Diagnosis of Pediatric Pneumonia Using Chest Radiographs

**DOI:** 10.7759/cureus.92596

**Published:** 2025-09-17

**Authors:** Justin Gillette, Michelle Lu, Thomas F Heston

**Affiliations:** 1 Medical Education and Clinical Sciences, Elson S. Floyd College of Medicine, Washington State University, Spokane, USA; 2 Internal Medicine, University of Washington School of Medicine, Seattle, USA; 3 Medical Education and Clinical Sciences, Washington State University, Spokane, USA; 4 Family Medicine, University of Washington, Spokane, USA

**Keywords:** ai in medical imaging, chatgpt, chest-radiography, diagnostic accuracy of ai, large language models, pediatric pneumonia

## Abstract

Introduction

Pneumonia remains a significant cause of morbidity and mortality in children globally. Chest radiographs (CXRs) are widely used to diagnose pediatric pneumonia; however, distinguishing between bacterial and viral etiologies on imaging is a diagnostically challenging task. Large language models (LLMs), particularly those integrated with vision capabilities, have shown promise in preliminary studies for interpreting CXR findings. However, the diagnostic performance of general-purpose LLMs without specialized medical training or add-ons remains poorly understood. This study examined whether such LLMs could independently and reliably distinguish between bacterial, viral, and normal CXRs in pediatric patients.

Methods

We evaluated four publicly available LLMs, such as ChatGPT o3, Claude 3.7 Sonnet, Gemini 2.5 Pro, and Grok 3, on a dataset of 44 pediatric CXRs confirmed by human readers to show bacterial pneumonia (n = 17), viral pneumonia (n = 13), or no abnormality (n = 14). Each image was analyzed twice by each LLM using a standardized prompt, resulting in a total of eight readings per image. Diagnostic accuracy was assessed relative to human expert consensus. Internal consistency was measured by comparing repeated interpretations. A prespecified adaptive stopping rule was employed based on performance futility criteria. Sample size calculations and statistical analyses were conducted using G*Power.

Results

Across all models and CXR types, the average diagnostic accuracy was 31%, consistent with chance-level performance in a three-choice classification task. Accuracy was highest for viral pneumonia (54%) and lowest for normal CXRs (18%). Internal consistency ranged from 46% to 71% across models, indicating unreliable performance. Concordance with human expert interpretation did not exceed 49% for any of the models. Futility criteria were met after 44 cases, prompting early termination of data collection.

Conclusion

General-purpose LLMs currently available to the public are not reliable diagnostic tools for pediatric pneumonia on chest radiographs. Their accuracy is low, particularly in ruling out disease, and their responses lack internal consistency. These findings highlight the risks associated with deploying such models in unsupervised clinical or consumer-facing settings. Future research should focus on purpose-built radiologic AI tools trained on diverse, clinically representative datasets and integrated with clinician oversight to ensure the safe and effective use of these tools.

## Introduction

Pneumonia is a leading cause of morbidity and mortality in pediatric populations worldwide, making prompt diagnosis essential [1]. Chest radiographs (CXRs) have been effective in ruling out pneumonia in children, which avoids unnecessary treatment with antibiotic therapy [2]. However, distinguishing between bacterial and viral pneumonia on chest X-rays has proven to be highly challenging, leading some studies to recommend that all children with radiologically confirmed pneumonia receive antibiotic treatment [3]. Even with these findings, clinicians continue to rely on CXRs to differentiate between bacterial and viral pneumonia, thereby guiding their treatment [4]. Since CXRs have a significant impact on the clinical management of pneumonia, it is crucial to enhance diagnostic accuracy.

Large language models (LLMs) like ChatGPT, with an “X-ray interpreter” add-on, have shown potential in identifying pathologies such as atelectasis, effusion, emphysema, pneumothorax, pneumonia, and masses on CXR [5]. ChatGPT with the add-on yielded varying results in identifying each pathology, but pneumonia showed the highest pathology accuracy at 91.0%, with a sensitivity of 76.2% and a specificity of 98.7% [5]. There has even been the development of radiology-dedicated LLMs, such as CXR-LLaVA, which have outperformed ChatGPT-4-vision and Gemini-Pro-Vision [6]. There will inevitably be an increase in the use of LLMs to help identify pathologies on CXRs. On October 29, 2024, even Elon Musk encouraged users to submit medical images to Grok for analysis [7]. Both clinicians and patients will increasingly have access to tools that can assist in diagnosis or validate existing findings. This highlights the importance of understanding the capabilities of AI models as well as the differences between them.

Independent evidence shows that general-purpose LLMs struggle with radiologic anatomy; in Part 1 radiologic-anatomy examinations for the Fellowship of the Royal College of Radiologists, ChatGPT-4 performed poorly, indicating significant limitations in recognizing normal radiological anatomy [8]. While prior studies have shown promising results using LLMs enhanced with specialized add-ons or radiology-specific tools, we aim to assess whether baseline, general-purpose models can independently provide valuable diagnostic insights without the need for additional modifications or integrations. To evaluate the efficacy of LLMs in diagnosing pneumonia from CXRs, we evaluated four leading LLMs, such as ChatGPT o3, Claude 3.7 Sonnet, Gemini 2.5 Pro, and Grok 3-for their ability to analyze pediatric CXRs categorized as showing bacterial pneumonia, viral pneumonia, or no abnormalities. The popularity of these LLMs increases the likelihood that patients could upload their CXRs to have a second opinion on their diagnoses. The LLMs will be evaluated for the accuracy of their diagnoses compared to those of human readers and their consistency when shown the exact CXR multiple times.

This study utilizes images from a public domain dataset that includes pediatric CXRs, which either display bacterial pneumonia, viral pneumonia, or a normal CXR [9,10]. These CXRs were obtained from retrospective cohorts of pediatric patients aged one to five years from Guangzhou Women and Children’s Medical Center in Guangzhou, China. These CXRs were interpreted to confirm a diagnosis and make treatment referrals [10].

The objective of this study was to evaluate the diagnostic accuracy and consistency of four leading general-purpose LLMs in classifying pediatric chest radiographs as bacterial pneumonia, viral pneumonia, or normal. The results of this study can help inform clinicians on how LLMs can be utilized or improved to enhance diagnostic accuracy. Additionally, patients should be aware of the potential for misdiagnosis when uploading their imaging and of which LLMs are the most consistent.

## Materials and methods

Study population

**Figure 1:**
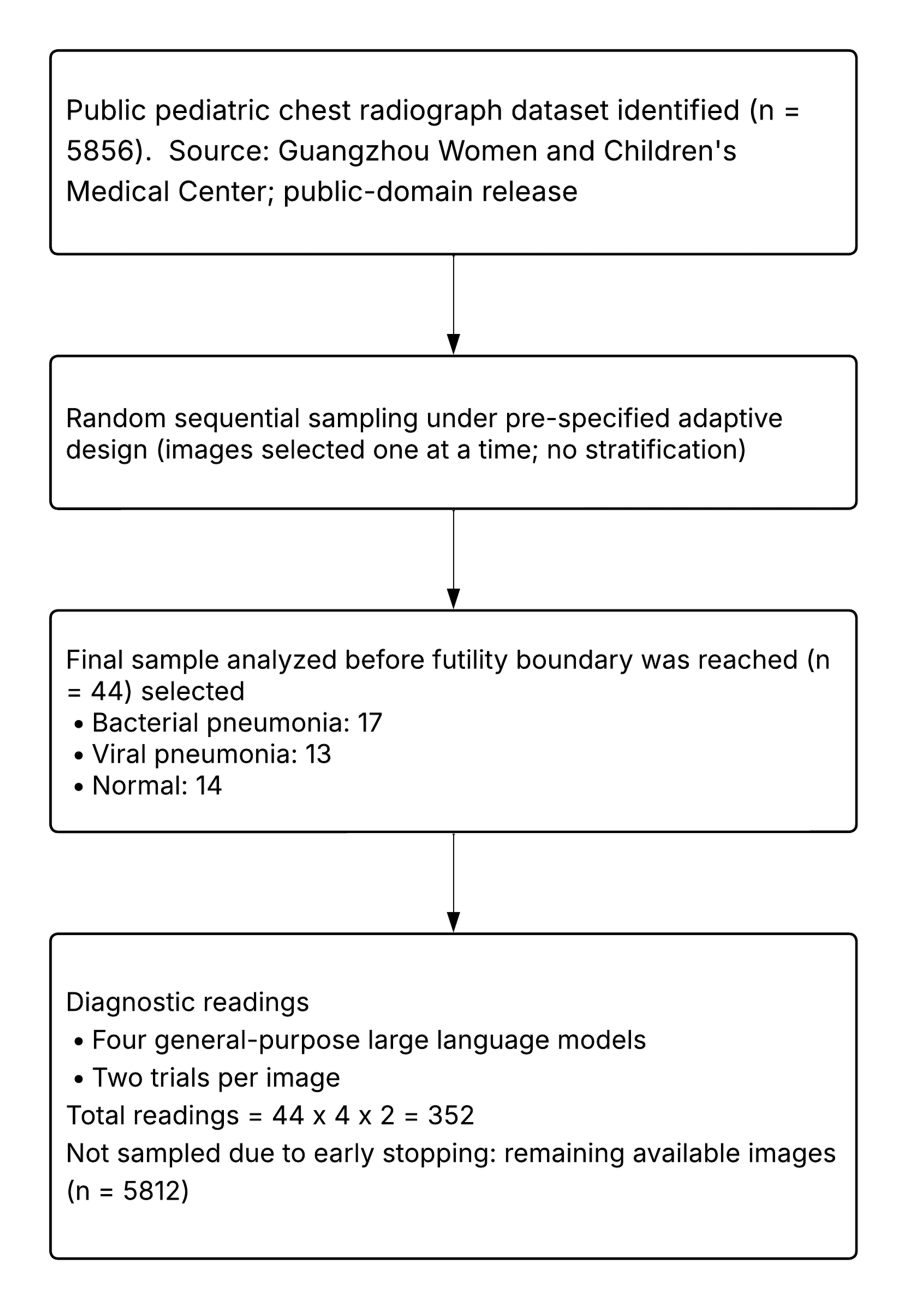
Standards for reporting diagnostic accuracy studies flow diagram A public pediatric chest radiograph dataset (n = 5,856) was identified. Images were randomly sampled sequentially under a prespecified adaptive design, and the analysis stopped when the futility boundary was crossed after 44 images (bacterial 17, viral 13, and normal 14). Each analyzed image was evaluated twice by four general-purpose large language models, yielding 352 total readings. The remaining available images (n = 5,812) were not sampled due to early stopping.

From a public dataset of 5,856 CXRs, 44 were randomly sampled and analyzed prior to meeting the adaptive futility boundary. Inclusion criteria, including patients aged one to five years, anterior-posterior image view, sufficient quality, and expert-confirmed diagnosis, were predefined by the public domain dataset curators. These images were evenly distributed across three diagnostic categories: bacterial pneumonia (n = 17), viral pneumonia (n = 13), and normal (n = 14). Each image was independently analyzed twice by four general-purpose LLMs, resulting in 352 total diagnostic interpretations (Figure 1). As each CXR was interpreted eight times, with non-unanimous predictions being common, we did not assign a single predicted diagnosis per image and did not compute standard cross-tabulation matrices. All models received the same standardized prompt, without prefix, suffix, or role assignment, consistent with prior studies demonstrating that prompt structure significantly affects diagnostic output from LLMs [8]. The full prompt was: “Look at this image carefully. Analyze it thoroughly. This is a CXR of a pediatric patient with suspected pneumonia. Based on the CXR, is the pneumonia bacterial or viral? Or is the CXR normal? Respond in one word: bacterial, viral, or normal.”

Statistical analysis

Sample size determinations were carried out in G*Power, version 3.1.9.7 (Heinrich-Heine-Universität Düsseldorf, Düsseldorf, Germany) [11]. Three analyses yielded N = 122 for test-retest consistency (Bowker’s marginal-homogeneity χ²; df = 3; w = .30; α = .05; 1-β = .80), N = 48 for AI-human concordance (χ² goodness-of-fit; df = 1; w = .408; α = .05; 1-β = .80), and N = 24 for comparing accuracy across the four programs via repeated measures ANOVA (f = .25; α = .05; 1-β = .80; measurements = 4; ρ = .50; ε = 1). The largest required sample size (N = 122) was chosen to ensure adequate power for all planned analyses. An adaptive trial design was employed, with one interim analysis scheduled after approximately one-third of the target sample had been evaluated [12,13]. Prespecified futility criteria were defined such that if the AI models' strict accuracy on the interim cohort remained within 5% of chance (i.e., 33%) and there was a low likelihood of achieving a clinically meaningful accuracy (>50%) with further data, then data collection would be stopped early. This approach minimized unnecessary evaluation of additional CXRs once evidence of futility was established. Although model responses were required to conform to a single-word format (“bacterial,” “viral,” or “normal”), no responses were excluded due to formatting issues; all outputs in the final dataset were interpretable and required no post hoc disambiguation. Although diagnostic categories were not perfectly balanced, case selection was based on image quality, patient age, and confirmed diagnosis rather than predetermined quotas, preserving the real-world nature of the dataset. Confidence intervals for diagnostic accuracy were calculated using the Wilson score interval without continuity correction [14].

Compliance with reporting standards

This study adhered to the Standards for Reporting Diagnostic Accuracy Studies (STARD 2015) guidelines for reporting diagnostic accuracy research involving retrospective imaging data [15]. A flow diagram and structured reporting elements were used to enhance transparency and reproducibility.

## Results

A total of 44 pediatric chest radiographs were analyzed between May and June 2025, after which the adaptive trial’s prespecified futility criteria were met, and data collection was stopped early.

Overall diagnostic accuracy

Across all four AI engines, the strict accuracy - defined as both model responses per image matching the human reference standard - averaged 31%, closely matching random-guess performance for a three-category task (chance = 33%). Individual model accuracies were 23% for Grok 3 (95% confidence interval (CI): 15%-33%), 27% for ChatGPT o3 (95% CI: 19%-37%), 45% for Claude 3.7 Sonnet (95% CI: 35%-55%), and 30% for Gemini 2.5 Pro (95% CI: 21%-40%; all CIs calculated using Wilson score method). One-way repeated measures ANOVA revealed no significant difference in accuracy between models (F(3, 129) = 2.178, p = 0.093). A statistical comparison of accuracies revealed significant differences between viral and normal pneumonia (z = 5.52, p < 0.001) and between viral and bacterial pneumonia (z = 4.46, p < 0.001). In contrast, the difference between bacterial and normal pneumonia was not significant (z = 1.39, p = 0.165).

AI-human concordance

Concordance with human radiologists was uniformly poor, with none of the models achieving better than 49% agreement and an overall average of 31%. χ² goodness-of-fit testing confirmed that this concordance did not differ significantly from chance performance (χ² = 0.819, df = 1, p = 0.366). The overall accuracy was low at 31% with a 95% CI of 25%-38%. For normal CXR findings, the accuracy was 16% (8%-28%); for viral, 54% (40%-67%); and for bacterial, 27% (17%-37%) (Table 1).

**Table 1:** Accuracy of LLMs by chest radiograph (CXR) type Accuracies of LLMs are given with 95% CIs. Accuracy refers to strict agreement between model output and human-confirmed reference standard.

CXR type	Accuracy (95% CI)	Interpretation
Normal	16% (8%–28%)	Substantially below chance; suggests unreliable performance in ruling out pathology
Viral	54% (40%–67%)	Slightly above chance; limited discriminative capability
Bacterial	27% (17%–37%)	Below chance; inconsistent differentiation from other etiologies
Overall	31% (25%–38%)	Within the range of random chance

Internal consistency

Test-retest consistency was evaluated using Bowker's marginal-homogeneity test (χ² = 2.622, df = 3, p = 0.454). While this indicated no systematic bias in the direction of disagreements between first and second readings, overall agreement was only 60.2%, demonstrating substantial inconsistency in model responses. Individual model consistency ranged from 46% to 71%, which is insufficiently reliable for clinical use.

Adaptive stopping

Interim analyses demonstrated that even under overly optimistic performance assumptions, final accuracy could not have risen appreciably above chance levels. Accordingly, the adaptive design’s stopping rule for futility was satisfied at n = 44, obviating the need to reach the initially planned N = 122 evaluations.

These findings indicate that baseline, general-purpose LLMs in their current form perform at chance levels for differentiating bacterial versus viral pneumonia (or normal) on pediatric CXRs and lack sufficient consistency to be considered reliable diagnostic aids.

## Discussion

The results of this study indicate a limited concordance between LLMs and human readers when interpreting CXRs. In addition, the LLMs showed low consistency in their readings when presented with the same CXR a second time. In a study by Yao et al., pediatric CXRs with confirmed pneumonia diagnoses were evaluated by four radiologists to assess their diagnostic performance [16]. The four radiologists (two attendings and two residents) in this study achieved a recall (sensitivity) range of 0.81-0.95 for normal CXRs, 0.49-0.71 for bacterial pneumonia, and 0.15-0.21 for viral pneumonia [16]. In contrast, all four LLMs evaluated in this study - general-purpose models without medical fine-tuning or domain-specific training - demonstrated low accuracy, even when identifying normal CXRs.

Their inconsistent responses when asked to reassess the same images suggest a lack of diagnostic confidence and stability. This variability raises concerns about susceptibility to bias, particularly if users repeatedly prompt the model until it produces an answer that aligns with their preconceived diagnosis. Given the models’ poor concordance with human readers and lack of internal consistency, there is a significant risk in relying on them independently, especially by users who may not fully understand the limitations and appropriate use of these tools.

The notably higher accuracy for viral pneumonia (54%) compared with bacterial pneumonia (26%) and normal CXRs (18%) suggests that these general-purpose LLMs may have differential pattern recognition capabilities across diagnostic categories. Viral pneumonia typically presents with bilateral, diffuse, or interstitial patterns that may be more readily identifiable by vision-enabled models compared to the focal consolidations characteristic of bacterial pneumonia. The inferior performance in identifying normal CXRs (18%) is concerning from a clinical perspective, as this represents the models' inability to rule out disease - a critical function in screening and diagnostic workflows. This pattern may reflect training data biases, where viral pneumonia images might be more prevalent in publicly available datasets, or it could indicate that the diffuse, bilateral patterns of viral disease align better with the pattern recognition algorithms underlying these models. The low accuracy for normal CXRs also suggests that these models struggle with the nuanced task of distinguishing subtle normal variations from pathology. This task requires sophisticated clinical judgment developed through extensive training and experience by human radiologists. These findings highlight the complexity of medical image interpretation and underscore the need for purpose-built, medically trained AI systems to achieve reliable diagnostic performance.

Since these publicly available LLMs cannot read as accurately as human radiologists, they would not provide much value if used alone. AI has been proposed as a potential solution to enhance access to medical imaging in low- and middle-income countries [17]. Many physicians ordering imaging may be tempted to use publicly available LLMs because they are easily accessible, have minimal barriers to use, and can quickly yield results. However, the LLMs used in this study are not suitable for practical or clinical use on their own, as their low accuracy significantly limits their reliability and effectiveness. Even if these LLMs are used with radiologist supervision, previous studies have shown that AI predictions with significant errors can lead to adverse treatment effects, with radiologists struggling to differentiate accurate and inaccurate outputs [18]. This highlights the need for accurate AI models that are specifically trained in medical imaging. Further research is needed to determine the most effective way to integrate AI into radiologists' workflows, enhancing diagnostic accuracy and efficiency while minimizing the addition of complexity or burden to their work.

While radiologists will be aware of the limitations of LLMs in interpreting medical imaging, the public will still be interested in using easily accessible LLMs to obtain a second opinion on their diagnoses. Many electronic medical record systems now grant patients immediate access to their imaging and lab results, often before a physician has reviewed them. Increasingly, patients prefer this real-time release of information, even if it has not yet been interpreted by a healthcare practitioner [19]. When patients gain access to their CXRs before a physician interprets them, many may experience anxiety and seek early interpretations from publicly available LLMs. This practice is concerning because LLMs could heighten patient distress with false positives or provide a false sense of reassurance in the case of false negatives. Since many patients are unaware of the limitations and low diagnostic reliability of AI, LLM-generated results may cause them to question or distrust the radiologist’s interpretation that they later receive.

The potential for misdiagnosis, misplaced trust, and unclear accountability demonstrates why these LLMs are not ready for patient care. Overreliance on AI tools for interpreting chest X-rays may give clinicians a false sense of diagnostic certainty, particularly for conditions such as bacterial pneumonia. This could lead to shortcuts in clinical reasoning, such as forgoing additional testing due to the convenience of a rapid AI-generated interpretation. Moreover, the issue of accountability remains unresolved. If an LLM produces an incorrect diagnosis, it is unclear whether responsibility lies with the clinician or the developers of the tool. These concerns highlight the urgent need for clear regulatory standards and rigorous validation before such models are used in patient care. Clinicians must understand both the limitations of these tools and the boundaries of their legal and ethical responsibility. Given the rapid advancement of AI models, evolving ethical concerns will require that regulations and standards be continually reassessed to safeguard patients and promote effective clinical practice [20].

Recent work has emphasized the potential role of agentic AI systems - autonomous agents that can plan, reason, and act within defined clinical boundaries - to improve diagnostic workflows in radiology [21]. While our study focused on general-purpose LLMs operating in passive diagnostic mode, the structured deployment of agentic AI may eventually address many limitations identified here. For instance, such systems could incorporate contextual clinical data, manage uncertainty by deferring to human experts, and engage in multistep diagnostic reasoning. However, realizing this potential will require robust governance, including privacy safeguards, interoperability standards, continuous performance monitoring, and phase-wise clinical integration. The radiology community must proactively evaluate and validate these tools to responsibly harness their benefits while safeguarding patient care.

Limitations

Several limitations should be considered in this study. A key limitation is the lack of transparency regarding the training data used for each LLM. Without knowing the specific images or datasets on which the models were trained, it is difficult to interpret the differences in their performance or understand the underlying factors contributing to their results. Even in authorized medical AI software, most products do not publish information on training data collection and population characteristics [22]. This uncertainty limits our ability to evaluate the training data and raises concerns about how well these models will generalize to different patient populations or clinical settings. LLMs function as black boxes, offering no insight into their decision-making processes [23]. To effectively compare LLMs to human radiologists, it is essential to understand the reasoning processes behind each diagnosis and how these processes may differ between models and humans. Another limitation of this study is that the LLMs were not evaluated in a real clinical environment. In real clinical practice, CXRs are not interpreted in isolation. Radiologists typically incorporate a range of complementary clinical information, such as patient history, physical examination findings, laboratory results, and prior imaging studies, to arrive at a diagnosis. This context is critical because many radiographic findings are nonspecific and require clinical correlation to determine their significance. By contrast, the LLMs in this study analyzed the CXR without access to any supporting clinical data, which does not reflect the way medical imaging is interpreted in real-world settings.

In addition, the dataset used in this study was sourced from a single institution, which introduces more limitations [24]. Imaging protocols, patient demographics, disease prevalence, and equipment settings can vary widely across hospitals, regions, and populations. As a result, the performance of these models on this dataset may not generalize to other clinical environments. This raises concerns about the external validity and real-world applicability of the findings, especially in more diverse or resource-limited settings where imaging conditions and patient profiles may differ substantially.

## Conclusions

The results of this study show that publicly available LLMs in their current state should not be used to evaluate pediatric CXRs for pneumonia. Their diagnostic accuracy remains significantly lower than that of human radiologists, and the low concordance between models indicates limited reliability and internal consistency in their diagnostic reasoning. If LLMs are to be used in a clinical setting, there must be extensive oversight by a radiologist in these early stages. Both clinicians and patients must be aware of the current limitations of these models and avoid relying on them as standalone diagnostic tools. Future work should focus on advancing AI tools that are specifically designed and trained for radiologic applications. These models should be developed using diverse and representative datasets that accurately reflect various patient populations to ensure broad generalizability. As LLMs continue to improve, their integration into clinical workflows must be accompanied by rigorous oversight to ensure the safe and effective use of these tools in patient care.
